# Hematinic and Iron Optimization in Peri-operative Anemia and Iron Deficiency

**DOI:** 10.1007/s40140-021-00503-z

**Published:** 2022-01-19

**Authors:** Lachlan F. Miles, Toby Richards

**Affiliations:** 1 Department of Critical Care, Faculty of Medicine, Dentistry and Health Sciences, The University of Melbourne, Melbourne, Australia; 2 Department of Anaesthesia, Austin Health, Melbourne, Australia; 3 Division of Surgery, Faculty of Health and Medical Science, The University of Western Australia, Perth, Australia

**Keywords:** Anemia, Iron Deficiency, Patient Blood Management, Perioperative Medicine

## Abstract

**Purpose of Review:**

Preoperative anemia is independently associated with worse postoperative outcomes following cardiac and noncardiac surgery. This article explores the current understanding of perioperative anemia and iron deficiency with reference to definition, diagnosis, and treatment.

**Recent Findings:**

Iron deficiency is the most common cause of anemia. It can arise from reduced iron intake, poor absorption, or excess iron loss. Inflammation throughout the preoperative period can drive iron sequestration, leading to a functional deficiency of iron and the development of what was referred to until recently as the “anemia of chronic disease.” Current best practice guidance supports the routine administration of preoperative intravenous iron to treat anemia despite limited evidence. This “one size fits all” approach has been called into question following results from a recent large, randomized trial (the PREVENTT trial) that assessed the use of a single dose of intravenous iron compared to placebo 10–42 days before major abdominal surgery. Although there were no improvements in patient-centered outcomes apparent during the initial hospital stay, secondary endpoints of this trial suggested there may be some late benefit after discharge from the hospital (8 weeks postoperatively). This trial raises questions on (1) the mechanisms of iron deficiency in the perioperative patient; (2) the need to reassess our opinions on generic anemia management; and (3) the need to address patient outcomes after discharge from hospital.

**Summary:**

Despite the known associations between preoperative anemia (particularly iron deficiency anemia) and poor postoperative outcome, recent evidence suggests that administering intravenous iron relatively close to surgery does not yield a tangible short-term benefit. This is made more complex by the interplay between iron and innate immunity. Iron deficiency irrespective of hemoglobin concentration may also impact postoperative outcomes. Therefore, further research into associations between iron deficiency and postoperative outcomes, and between postoperative anemia, delayed outcomes (hospital readmission), and the efficacy of postoperative intravenous iron is required.

## Introduction

Anemia is defined as “a condition in which the number of red blood cells is insufficient to meet the body’s physiologic needs” [1], and it is defined conventionally using the World Health Organization (WHO) laboratory reference range for hemoglobin concentration [Hb], namely, < 120 g/L for women or < 130 g/L for men. Anemia is a common finding during the preoperative assessment of patients scheduled for major surgery, occurring in 10–15% of patients requiring elective orthopedic and up to 60% of patients requiring colorectal surgery [2]. Anemia is relevant to the perioperative physician for two reasons: (1) Preoperative anemia is independently associated with worse postoperative patient outcomes, and (2) preoperative anemia is a therapeutic target.

The first has become a recognized point of acceptance. However, confusion and equipoise exist around the second, with questions asked about diagnosis and management of iron deficiency and how different patient populations should be managed in the perioperative period.

In this article, we review the current understanding of anemia and iron deficiency in the surgical setting. Additionally, we comment on the recent PREVENTT trial, where patients undergoing elective major open abdominal surgery were randomly allocated to receive preoperative (10–42 days before surgery) intravenous iron or placebo in a double-blind, parallel-group study design [3••]. PREVENTT is the largest placebo-controlled trial of pre-operative intravenous iron to date. The reported results raise important questions about intravenous iron administration in surgical populations.

## Defining Anemia and Outcomes After Major Surgery

### Anemia and Postoperative Outcomes

The association between preoperative anemia and increased postoperative complications has been reported in several different surgical populations. In a landmark 2011 study, Musallam et al. analyzed 227,425 patients who had undergone major noncardiac surgery from the American College of Surgeons National Surgical Quality Improvement (NSQIP) database [4]. Not only did these authors demonstrate that patients with preoperative anemia were more likely to suffer from additional major comorbidities, but also that once these confounding factors were corrected for, there were significant associations between preoperative anemia and mortality (OR 1.42, 95% CI 1.31–1.54) and composite morbidity (OR 1.35, 95% CI 1.3–1.4). Further data from this study cohort published by Clevenger et al. showed a linear association between a falling trajectory of preoperative hematocrit and worsening perioperative morbidity and mortality [5]. A subsequent meta-analysis by Fowler et al., incorporating 949,445 patients from 24 studies in cardiac and noncardiac surgery, reported an association between preoperative anemia and postoperative mortality (OR 2.9, 95% CI 2.3–3.68, *p* < 0.001), acute kidney injury (OR 3.75, 95% CI 2.95–4.76, *p* < 0.001), and infection (OR 1.93, 95% CI 1.17–3.18; *p* = 0.01) [6]. These associations were validated in a prospective audit of 19,033 patients undergoing cardiac surgery in the UK, where Klein et al. reported that preoperative anemia was associated with increased mortality (OR 1.42, 95% CI 1.18–1.71; *p* < 0.001) and hospital stay (geometric mean ratio 1.15; 95% CI 1.13–1.17; *p* < 0.001) [7].

In summary, preoperative anemia is associated with adverse postoperative patient outcomes in a “dose-dependent” manner, as supported by retrospective and prospective studies in most surgical subgroups, including patients having cardiac [6] colorectal [8, 9], gynecological [10], and vascular surgery, as well as in the elderly surgical patient cohort [11]. Despite the apparent strength of evidence outlined above, it is essential to recognize that these data may be confounded by chronic and comorbid disease [12]. Nevertheless, preoperative hemoglobin optimization has become not only a cornerstone of Patient Blood Management programs but perhaps also the *raison d'etre* [13–17] and is therefore included in prehabilitation strategies.

### Alternative Definitions of Anemia

Laboratory values for the normal reference range of hemoglobin and that which defines anemia may vary. The normal reference range typically reflects the mean and two standard deviations of values within a “healthy population,” which according to the Clinical Laboratory Standards Institute guidelines can be based on a minimum sample size of only 120 participants [18, 19]. The World Health Organization (WHO) definitions may be outdated and biased against female patients, potentially including women with iron deficiency or anemia in the “normal population” range [20]. This is supported by a retrospective study of 1,388 women undergoing cardiac surgery by Blaudszun et al. In this study, women with a preoperative hemoglobin concentration between 120 and 130 g/L (within the normal range for women, but regarded as anemic for men) showed that this population of women when compared to women with a preoperative hemoglobin concentration ≥ 130 g/L were more likely to receive a blood transfusion (68.6% vs. 44.5%; RR 1.5, 95% CI 1.4–1.7; *p* < 0.001) and had an increased length of hospital stay (8 [IQR 6–12] days vs. 7 [IQR 6–11] days; *p* = 0.0159) [21]. This is further supported by a large audit of 19,033 cardiac surgical patients in the UK, which demonstrated a nadir for the length of stay and for mortality within 30 days after cardiac surgery at an even higher hemoglobin concentration—140 g/L—for both men and women [7]. In major abdominal surgery, Miles et al. demonstrated that women with a preoperative hemoglobin concentration between 120 and 130 g/L had a greater incidence of postoperative complications (16% vs. 11%; *p* = 0.017) and an increased length of hospital stay (3 [IQR 1–6] days vs. 2 [1−5] days; *p* = 0.017) compared to those women with normal hemoglobin concentration, ≥ 130 g/L. Consequently, consensus statements now advocate for a unified perioperative anemia threshold of < 130 g/L for women and men [15].

## Cause and Consequence of Iron Deficiency

### The Heme and Non-heme Roles of Iron

Iron is universally acknowledged as a key component of hemoglobin. However, only about two-thirds of bodily iron is involved in oxygen carriage, either by hemoglobin or myoglobin in muscles [22]. Additionally, iron is also important for many enzymatic processes across multiple tissue sites, including cytochrome function in mitochondria, neurotransmitter production, and immune function. Consequently, iron is essential for oxygen transport and oxygen utilization, buffering fluctuations in intracellular oxygen concentration, facilitating aerobic metabolism and ATP generation, and scavenging nitric oxide and other free radicals [23]. Perhaps the best data for the consequences of iron deficiency (as opposed to anemia) are derived from the heart failure literature, where large, randomized trials [24] and subsequent meta-analyses [25, 26] have demonstrated that iron supplementation in patients with iron deficiency is associated with reductions in all-cause mortality and hospitalization in patients with heart failure, independent of hemoglobin concentration.

### Defining Perioperative Iron Deficiency

Routine blood tests typically comprise some combination of serum iron, transferrin (a plasma glycoprotein that transports iron), transferrin saturation (Tsat), ferritin (an intracellular protein that stores and controls the release of iron), and C-reactive protein (CRP), with differing definitions and thresholds reported in the literature [27, 28]. Serum ferritin is the most sensitive marker of iron deficiency and is indicative of low iron stores. However, ferritin is an acute-phase reactant that is commonly elevated preoperatively in the surgical population [29]. This is illustrated by a systematic review of 38 studies where > 1,000 patients with iron deficiency diagnosed on bone marrow staining had average serum ferritin of 82.4 μg/L (range 34–159 μg/L) within the normal reference range (> 30 μg/L) for most laboratories [30]. This study highlights a critical concept: that in isolation, serum ferritin above the lower limit of the “normal” reference range (> 30 μg/L) cannot be considered an accurate reflection of iron availability as iron deficiency exists in two forms [31]: absolute iron deficiency—a true absence of stored iron; and functional iron deficiency—an inability to access adequate iron stores.

**Fig. 1:**
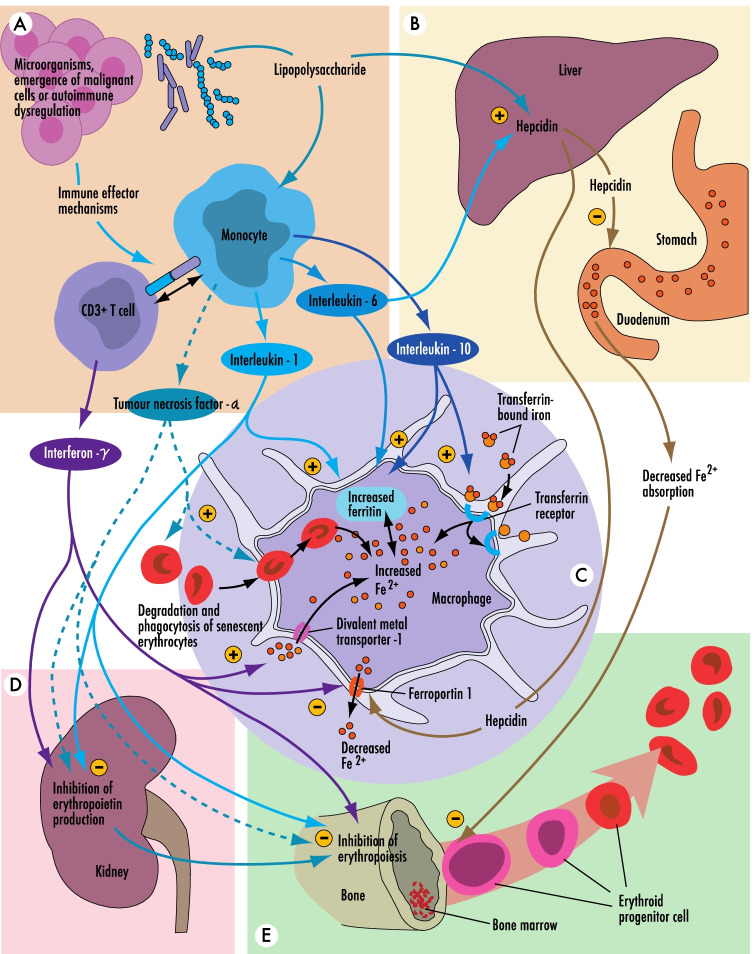
Overview of normal iron homeostasis demonstrating the interplay between innate immunity (A), the hepcidin-ferroportin axis (B), movement of iron across the basement membrane of an iron bearing cell (in this case, a macrophage) (C), the effects of inflammation on erythropoietin production (D), and erythropoiesis in the bone marrow (E)

Absolute iron deficiency accompanies nutritional deficiency, inadequate iron absorption, or because of excess blood loss leading to a total body depletion of iron stores. Functional iron deficiency is due to iron sequestration and the inability to transport iron to sites of need. The latter is commonly in response to inflammation, with interleukin-6 (IL-6)–mediated upregulation of the iron regulatory protein, hepcidin [32, 33], which inhibits iron transport across basement membranes through the downregulation the iron transport protein ferroportin-1. Elevated hepcidin states are seen in a variety of chronic disease states, often characterized by underlying inflammation, including malignancy, autoimmune disorders, heart failure, diabetes, and renal impairment, with resultant failure of iron transport across basement membranes, iron sequestration, and a state of functional iron deficiency (Figure 1) [34]. Chronic functional iron deficiency and the direct impacts of inflammation on the bone marrow lead to reduced erythropoiesis and “anemia of chronic disease” [35, 36••].

Observational studies examining the underlying causes of preoperative anemia estimate that the combination of absolute and functional iron deficiency accounts for most cases of preoperative anemia [27, 28]. However, considerable variation exists in the definition of iron deficiency anemia; but consensus has arisen in most perioperative guidelines [13, 15, 17, 37] that a reduced hemoglobin with a combination of serum ferritin < 100 μg/L and/or Tsat < 20% is indicative of iron deficiency anemia (Table 1).

**Table 1:** Studies examining the association between iron deficiency and postoperative outcome in patient cohorts both with and without anemia

Reference	Study design	Population	*n*	Definition of iron deficiency	Outcome
Piednoir 2013 [37]	Prospective, single center	Cardiac surgery	• 100 (63 iron replete, 37 iron deplete) • Anemia and non-anemia	• Ferritin < 80 μg/L • Ferritin 80–150 μg/L and Tsat < 20% • Tsat < 20% and sTfR/log ferritin ≥ 0.7	• Increased morbidity: transfusion • Increased morbidity: postoperative fatigue
Wilson 2017 [41]	Retrospective, single center	Colorectal cancer surgery	• 339 (176 iron replete, 163 iron deplete) • Anemia and non-anemia	• TSAT < 20%	• Increased morbidity: postoperative complication
Miles 2018 [38]	Retrospective, single center	Cardiac surgery (CABG or single valve)	• 277 (168 iron replete, 109 iron deficient) • Non-anemia only	• Ferritin < 100 μg/L • Ferritin 100 – 300 μg/L and TSAT < 20%	• Increased morbidity: length of hospital stay • Increased morbidity: DAH-30
Miles 2019 [42]	Retrospective, single center	Colorectal cancer surgery	• 141 (36 iron replete, 105 iron deficient) • Non-anemia only	• Ferritin < 100 μg/L • Ferritin 100–300 μg/L and TSAT < 20%	• Nil; exploratory study for identification of relevant endpoints
Rössler 2020 [39]	Pseudo-prospective, single center	Cardiac surgery	• 730 (574 iron replete, 156 iron deficient) • Anemia and non-anemia	• Ferritin < 100 μg/L	• Increased mortality • Increased morbidity: SAE • Increased morbidity: length of stay • Increased morbidity: transfusion
Immohr 2021 [40•]	Pseudo-prospective, single center	Cardiac surgery (CABG or single valve)	• 395 (113 iron replete, 265 iron deficient) • Anemia and non-anemia	• Ferritin < 100 μg/L • Ferritin 100–300 μg/L and Tsat < 20%	• Nil

*CABG*, coronary artery bypass grafting; *DAH-30*, days alive and at home on postoperative day 30; *sTfR*, soluble transferrin receptor; *Tsat*, transferrin saturation; *SAE*, serious adverse event

### Preoperative Iron Deficiency (Independent of Hemoglobin) and Postoperative Outcome

Relative to anemia, there are few observational studies examining iron deficiency (in non-anemic patients and irrespective of [Hb] status) and outcome following major surgery (Table 2). Observational studies in cardiac [38, 39, 40•, 41) and colorectal surgery [42, 43] populations report weak associations between iron deficiency and worse postoperative outcomes irrespective of hemoglobin concentration. However, the small and retrospective or pseudo-prospective nature (retrospective analysis of data collected prospectively for another purpose) of most of these studies mean that further work (ideally multicenter) is required to better define this relationship beyond that of the known association between anemia and poor postoperative outcome. Despite this relative lack of evidence, best practice guidelines and consensus statements recommend the preoperative treatment of non-anemic iron deficiency before major surgery [13, 15]; however, it is unlikely that iron studies are routinely performed in non-anemic patients.

**Table 2:** A basic diagnostic approach to pre-operative laboratory markers of iron status

Value	Iron replete	Absolute iron deficiency	Functional iron deficiency	Inadequate iron stores
Ferritin (μg/L)	≥ 100	< 30	100–300	< 100
Tsat (%)	≥ 20	Any	< 20	Any

*Tsat*, transferrin saturation

## Treatment of Perioperative Iron Deficiency and Anemia

Historically, intravenous iron, especially with the high molecular weight iron dextran, has been associated with a high risk of allergic reactions. However, the development of more stable carbohydrate-based intravenous iron preparations has markedly increased the safety of intravenous iron [44, 45]. Consequently, an increase in the prescription of these medications (i.e., iron carboxymaltose and iron isomaltoside) has been seen over the last decade, prompting some authors to question whether this intervention is being administered appropriately and cost-effectively [46]. Intravenous iron has important advantages over oral preparations:

1. Oral iron is absorbed in the duodenum, and any disease affecting the gut can impact absorption [47].
2. Oral iron has a high incidence of gastrointestinal side effects (up to 40% in some series) due to local toxicity in the enterocytes causing abdominal pain, constipation, or diarrhea that limit adherence with therapy [48, 49•].
3. Non-heme iron is poorly absorbed. Of the recommended daily dose of 65 mg of elemental iron (200 mg of iron sulfate), only 5–10 mg is absorbed per day. Consequently, assuming absolute iron deficiency (ferritin < 30 μg/L), the body will require at least 1000 mg to replenish stores and many months of therapy for full effect [50].

Accordingly, preoperative oral iron therapy is helpful as a preoperative optimization strategy only where there are months available before surgery, high patient compliance, absence of gut malabsorption, and the absence of inflammation [51].

In a randomized controlled trial comparing oral iron with intravenous iron [49•], 116 patients with anemia who were planned to undergo surgery for colorectal cancer were allocated randomly to receive oral iron (ferrous sulfate, 200 mg twice daily) or intravenous iron (ferric carboxymaltose, up to 2,000 mg, with dosing based on weight and hemoglobin concentration). The median duration of study iron treatment was 21 days (IQR 15–34 days) in both the oral iron and intravenous iron groups. Patients in the intravenous iron group had a significantly higher hemoglobin concentration at the time of surgery, with a median treatment hemoglobin concentration rise of 1.55 (IQR 0.93–2.58 g/dL) versus 0.5 (IQR −0.3 to 1.33 g/dL; *p* < 0.001) in the oral iron group. However, no differences were noted in the primary endpoint of perioperative blood transfusion.

Some concerns have been raised in the literature regarding the propensity for iron therapy to increase the risk of infection. A 2013 systematic review and meta-analysis by Litton et al. (2013) reported an increase in hemoglobin concentration and a decrease in transfusion requirement with intravenous iron; however, they also found a significant increase in the risk of infection (RR 1.33, 95% CI 1.1–1.64) [52]. This is biologically plausible, as the body will render itself iron deplete through the hepcidin-ferroportin axis at times of bacteremia as a means of depriving possible pathogens of metabolic fuel [53]. However, this signal has not been replicated in large retrospective [54] or prospective trials [55•]. Indeed, signals from retrospective observational studies of non-anemia iron deficiency [56] suggest that the inverse is also biologically plausible—that being iron deficient at the time of operation poses an increased risk of postoperative infection. Consequently, the data at present does not suggest that intravenous iron administered relatively close to surgery increases the risk of postoperative infection. However, data is notably absent concerning patients who have an active infection at the time of their surgery.

### Intravenous Iron Versus Placebo

A series of small interventional trials on the use of intravenous iron in patients with preoperative anemia reported improved hemoglobin concentration before surgery, relative to placebo, but with no reduction in blood transfusion [57–60]. In cardiac surgery, the best evidence for intravenous iron in isolation is a propensity-matched, retrospective cohort study [61] and a randomized trial that examined the effect of multi-component interventions (with the caveat that the placebo group had a greater number of high-volume transfusions, suggestive of surgical bleeding) [55•]. A systematic review performed in 2014 [62] and updated in 2019 [63••] highlighted the lack of evidence of benefit for the use of intravenous iron in noncardiac surgery for transfusion prevention and hemoglobin incrementation and suggested that further research was required. The subsequent “pre-operative intravenous iron to treat anemia before major abdominal surgery” (PREVENTT) trial aimed to address this knowledge gap [3••].

PREVENTT, the largest prospective randomized control trial performed to date, assessed patients with anemia 10–42 days before major elective abdominal surgery with randomization to intravenous iron (ferric carboxymaltose, 1000 mg) or placebo. The patient population included 487 patients with a median age of 66 (IQR 54–72) years; 55% of participants were women, with most patients having comorbid disease (American Society of Anesthesiologists Physical Status Class II [61.0%] or III [25.6%]). The study drug was administered (in a double-blind manner) a median of 15 (IQR 12–22 days) before major open abdominal surgery (including esophagectomy, gastrectomy, pancreatectomy, colectomy, hysterectomy). A composite primary outcome of death or blood transfusion was used, with important patient-centered secondary outcomes examined. PREVENTT showed that intravenous iron was not superior to placebo in reducing perioperative transfusion requirement (29% vs. 28%; *p* = 0.92) or when assessed by transfusion episodes (105 in the intravenous iron group vs. 111 in the placebo group). There was no difference in significant postoperative complications (24 [11%] vs. 22 [9%]; RR 0.89, 95% CI 0.52 to 1.55; p = 0.69), length of hospital stay (median [IQR]: 9 [7–14] days vs. 9 [5–14] days; *p* = 0.14), or mortality rate (1% vs. 1%; p = 1.0) for patients in the placebo group relative to intravenous iron group, respectively. These findings were unaffected by subgroup analysis (age, gender, baseline hemoglobin concentration, iron status) or per-protocol analysis (Table 3) [64••].

**Table 3:** Subgroup analyses from the PREVENTT trial, showing the results of the primary outcome analysis (blood transfusion or death within 30 days) when stratified by underlying baseline iron status

	Placebo *n*/*N* (%)		IV iron *n*/*N* (%)		Risk ratio (95% CI)		*p*-value^1^
**Ferritin (ng/mL)**							
< 30	17/69	(24.6)	14/75	(18.7)	0.76	(0.40 to 1.42)	0.33
30–100	17/63	(27.0)	20/53	(37.7)	1.40	(0.82 to 2.38)	
> 100	32/98	(32.7)	31/94	(33.0)	1.01	(0.67 to 1.51)	
**Tsat (%)**							
< 20	55/174	(31.6)	49/163	(30.1)	0.95	(0.69 to 1.31)	0.13
> 20	8/50	(16.0)	15/53	(28.3)	1.77	(0.82 to 3.81)	
**Ferritin and Tsat**							
Ferritin < 100 ng/ml **OR** Tsat < 20%	58/193	(30.1)	53/176	(30.1)	1.00	(0.73 to 1.37)	0.66
Ferritin 100 + ng/ml **AND** Tsat > 2%	8/37	(21.6)	12/46	(26.1)	1.21	(0.55 to 2.64)	
Ferritin < 100 ng/ml **AND** Tsat < 20%	31/113	(27.4)	30/115	(26.1)	0.95	(0.62 to 1.46)	0.49
Ferritin > 100 ng/ml OR Tsat > 20%	32/111	(28.8)	34/101	(33.7)	1.17	(0.78 to 1.74)	

^1^Interaction *p*-value

The results of the PREVENTT study were unexpected as the association of preoperative anemia with increased adverse postoperative outcomes was seemingly well established. Furthermore, the use of intravenous iron appeared an easy, safe, and plausible treatment that had been shown effective in selected study cohorts. PREVENTT, therefore, epitomizes the need for any good idea or opinion to be tested in the setting of a large, well-conducted randomized controlled trial setting before guidelines are made [17]. That said, PREVENTT is not without limitations (including a relatively short time span between intravenous iron administration and surgery, lack of dose adjustment to baseline hemoglobin concentration or patient weight), and it has stimulated considerable discussion in the literature, with several editorials devoted to the trial and its implications [65–69].

PREVENTT was designed to determine whether the association of anemia with adverse outcomes after major abdominal surgery could be reversed with intravenous iron. The data on these associations was for all-cause anemia. Like all 24 studies included in the meta-analysis by Fowler et al., preoperative iron deficiency was not defined [6]. Similarly, baseline iron studies were not part of routine clinical care in the country where PREVENTT was run [3••]. Consequently, PREVENTT assessed the effect of the intervention on iron deficiency by a predefined subgroup analysis using core laboratory measurement of serum ferritin and Tsat obtained at randomization. Overall, 71% of participants had a Tsat < 20%, and 55% had serum ferritin < 100 μg/L, the current consensus criteria for iron deficiency. Interestingly, of the 369 patients with this consensus definition of iron deficiency, the primary outcome of death or need for blood transfusion was identical (30.1%) in both the intravenous iron and placebo groups [64••]. Blood transfusion practice was standardized in PREVENTT as trial sites were selected after compliance with the National Health Service (NHS, UK) Blood and Transplant guidelines [2], with two independent audits during recruitment demonstrating that the average transfusion trigger was a hemoglobin concentration of 84 (IQR 75–92) g/L [3••]. These audits also showed that most patients undergoing major abdominal surgery were listed for surgery 15–30 days before the surgery proceeded and underwent formal preoperative assessment 10–16 days before surgery. The study drug was delivered a median of 15 days (IQR 12–22) preoperatively.

When interpreting PREVENTT, it is important to understand the question that the trial asked. The results of PREVENTT do not suggest that intravenous iron for the treatment of preoperative anemia be abandoned in all forms; instead, the trial concludes that there the possible benefits in the use of intravenous iron in all patients with anemia in the typical timelines before major abdominal surgery (particularly abdominal cancer surgery) were limited, and that those benefits might be replicated by giving intravenous iron in the immediate postoperative period (although this hypothesis is yet to be tested formally). Intravenous iron is indicated for the treatment of iron deficiency where oral iron is not tolerated or does not work. For those patients in PREVENTT who had a serum ferritin < 30 μg/L at enrolment (144 of the 487 participants included in the trial), intravenous iron would usually be indicated for reasons separate to perioperative patient blood management [50]. Theoretically, if approximately one in three patients present with preoperative anemia, and of those, one in three have absolute iron deficiency, then approximately 1 in 10 (30 patients in the PREVENTT cohort) should receive treatment irrespective of whether they were awaiting surgery. However, a key aspect of PREVENTT is that it found iron therapy not to be superior in this subgroup or any other means of defining iron deficiency anemia. Hence, it appears unnecessary to administer the treatment preoperatively if the objective is to reduce transfusion rates. Ironically, the results of PREVENTT are reassuring to the anesthesia and surgical community as they show that a patient does not require a hospital visit to treat preoperative anemia before relatively urgent elective. This is important, not only from a health economic perspective, but also from a patient and staff safety perspective given the current COVID-19 pandemic.

Two additional key points arise from PREVENTT: (1) it is not clear how to define iron deficiency in the preoperative patients with concomitant inflammation (i.e., CRP > 5 mg/L), and (2) there may be benefit from iron infusion to reduce readmissions for postoperative complications (particularly readmission > 8 weeks after surgery). Whether this is related to the peak incrementation effect (e.g., 6 weeks) on hemoglobin concentration and non-heme iron pathways (e.g., myoglobin, cytochrome enzymes) after the iron infusion or implies that iron infusions can be administered *at* or *after* surgery warrants further research.

The incrementation in hemoglobin concentration seen in PREVENTT was lower than expected, with a mean difference between intravenous iron and placebo of 4.7 g/L (95% CI 2.7–6.8 g/L) over a median period of 15 days before surgery. Anemia was corrected in only 21.1% of patients before surgery. In a study by Froessler et al. in which preoperative patients were treated with intravenous iron a median (IQR) of 8 (6–13) days before their surgery, hemoglobin concentration rose approximately 8 g/L from a baseline mean (SD) of 107 g/L (13 g/L) to 115 g/L (13 g/L) [60]. In the IVICA trial, where patients with colorectal cancer were randomized an average of 21 (15–34) days before surgery, the mean rise in hemoglobin concentration was 15.5 g/L with intravenous iron (and only 5.0 g/L with oral iron) [49•]. The heterogeneity in these trial results may reflect variations in the timing of administration before surgery, and perhaps more importantly the causality of anemia and iron deficiency in these different populations, those trials being predominantly colorectal surgery. In PREVENTT, a contributing factor to this variable response to iron infusion may have been that 71% of participants had a Tsat < 20%, 55% had serum ferritin < 100 μg/L, and only 30% had serum ferritin < 30 μg/L (Table 3). Furthermore, the interplay between inflammatory mediated iron sequestration, blood loss from gastrointestinal causes or chronic disease with bone marrow suppression are likely to be important factors to consider in assessing the response to iron and also the impact of this therapy on postoperative outcomes. Further experimental work to assess hepcidin levels and other diagnostic criteria for iron deficiency in PREVENTT is ongoing. The use of erythropoietin may additionally improve the efficacy of the intervention, as seen in patients scheduled for cardiac surgery [55•]. However, PREVENTT showed agreement with Spahn et al. that a generic preoperative anemia management intervention had no impact on patient outcomes; neither trial showed a beneficial impact on postoperative adverse events or length of intensive care unit or hospital stay after surgery [4].

**Fig. 2:**
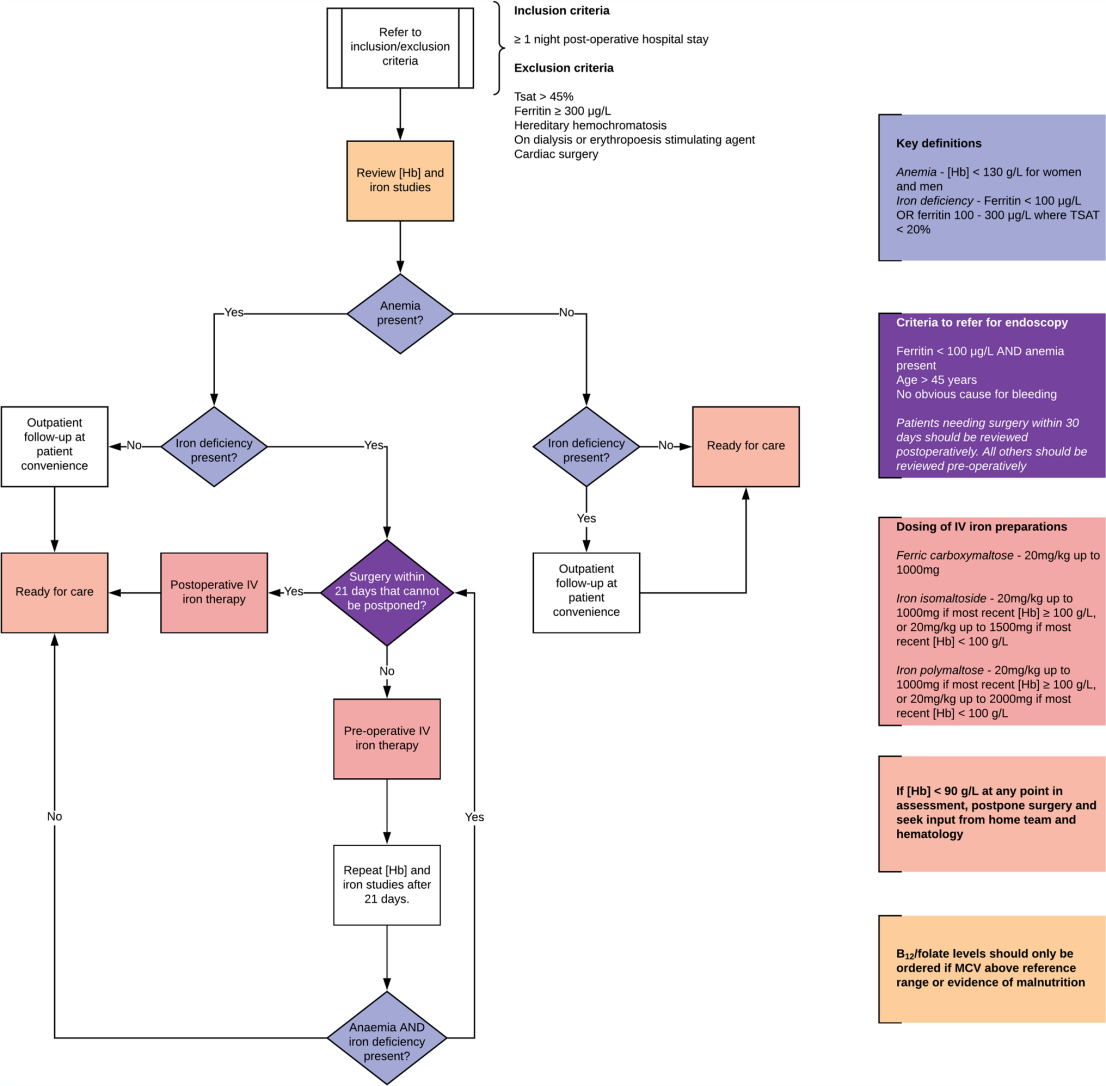
Hypothetical guidance for perioperative iron therapy for non-cardiac surgery in light of the results of the recent PREVENTT trial [3••]. Abbreviations: *[Hb]*, hemoglobin concentration

The finding in PREVENTT that patients allocated to the intravenous iron group were less likely to require readmission to hospital at 8 weeks (RR 0.61, 95% CI 0.4–0.9) and 6 months (RR 0.78, 95% CI 0.58–1.04) after surgery warrants further discussion. This observed difference was associated with a marked increase in the late postoperative hemoglobin concentration in the intervention group. However, this observation was not associated with increased days alive and out of hospital on postoperative day 30, nor an increase in patient-reported quality of life at 6 weeks or 6 months. Nevertheless, those patients who were anemic and had received intravenous iron who also had major blood loss during surgery appeared to have reduced associated complications after discharge. This raises questions regarding the optimal timing between intravenous infusion and surgery and whether patients would benefit from intravenous iron in the postoperative period. Intra-operative intravenous iron could also be considered, although efficacy in the event of major intra-operative hemorrhage is unknown. A hypothetical guideline for how and when perioperative intravenous iron might be delivered to elective abdominal surgery patients following the results of PREVENTT is shown in Fig. 2.

Postoperative anemia has largely been ignored, but the evidence that does exist is reported in Table 4. Oral iron therapy does not work in this setting due to the inflammatory-mediated elevation of hepcidin levels that block iron absorption from the gut. In a large analysis from the Kaiser Permanente Northern California network of 21 hospitals, which serves 4 million members, it was noted that blood transfusion use declined over the last decade of clinical practice with no associated increase in in-hospital complications or mortality [70]. However, one in four patients were discharged with Hb < 100 g/L. Those who failed to recover their Hb and remained anemic were more likely to be re-admitted to hospital in the immediate 30 days following hospital discharge. At follow-up two-thirds remained anemic at 6 months. Similar findings were also seen in data from the Cleveland clinic network, with 72% of the 152,757 patients included being discharged with anemia [71]. Of those that were anemic, 20% were re-admitted to hospital within 30 days. Discharge anemia was associated with a severity-depending increase in odds for 30-day hospital readmission compared with those without anemia: mild anemia, 1.74 (95% CI 1.65–1.82); moderate anemia, 2.76 (95% CI 2.64–2.89); and severe anemia, 3.47 (95% CI 3.30–3.65), *p* < 0.001. These data suggest that postoperative anemia—particularly discharge hemoglobin concentration—is strongly associated with poor postoperative outcome.

**Table 4:** Studies comparing postoperative intravenous iron to placebo or oral iron for the treatment of anemia after major surgery

Reference	Study design	Population (*n*)	Intervention (*n*)	Comparator (*n*)	Outcome
Madi-Jebara 2004 [71]	Double blinded RCT, single center	Cardiac surgery (*n* = 61)	• Intravenous iron sucrose (*n* = 30)	• Placebo (*n* = 31)	• No difference in [Hb] • No difference in transfusion
Karkouti 2006 [72]	Double blinded RCT, single center	Cardiac surgery (*n* = 26)	• Intravenous iron sucrose (*n* = 13)	• Placebo (*n* = 13)	• No difference in [Hb] • No difference in transfusion
Bisbe 2014 [73]	Single blinded RCT, single center	Total knee arthroplasty (*n* = 122)	• Intravenous ferric carboxymaltose (*n* = 60)	• Oral ferrous sulfate (*n* = 62)	• Higher Hb response at POD 30 • No difference in QoL
Litton 2016 [74] ‘IRONMAN’	Double blinded RCT, multicenter	ICU patients (87% surgical) (*n* = 140)	• Intravenous ferric carboxymaltose (*n* = 70)	• Placebo (*n* = 70)	• Higher [Hb] at hospital discharge • No difference in transfusion
Khalafallah 2016 [75]	Open label RCT, pseudo-multicenter	Elective major orthopedic, abdominal or genitourinary surgery	• Intravenous ferric carboxymaltose (*n* = 103)	• Standard care (*n* = 98)	• High [Hb] at POD 30 • Decreased transfusion
Kim 2017 [76] ‘FAIRY’	Double blinded RCT, multicenter	Gastric cancer surgery (*n* = 454)	• Intravenous ferric carboxymaltose (*n* = 228)	• Placebo (*n* = 226)	• Higher [Hb] at POD 84 • No difference in transfusion • No difference in QoL
Xu 2019 [77]	Single blinded RCT, single center	Cardiac surgery (*n* = 150)	• Intravenous iron sucrose (*n* = 75)	• Placebo (*n* = 70)	• Higher [Hb] at POD 14 • No difference in transfusion • No difference in morbidity/mortality
Muñoz 2014 [78]	Retrospective observational (propensity matched)	Total knee and hip arthroplasty (*n* = 364)	• Intravenous iron sucrose (*n* = 184)	• Standard care	• Higher [Hb] at POD 7 • Reduced transfusion • Reduced cost
Jeong 2014 [79]	Retrospective before-and-after	Gastric cancer surgery (*n* = 142)	• Intravenous ferric carboxymaltose (*n* = 68)	• Standard care (*n* = 74)	• Higher [Hb] at POD 180
Kim 2018 [80]	Retrospective before-and-after (propensity matched)	Total hip arthroplasty (*n* = 300)	• Intravenous ferric carboxymaltose (*n* = 150)	• Standard care (*n* = 150)	• Higher Hb response at POD 42 • Lower transfusion

*RCT*, randomized controlled trial; *[Hb]*, hemoglobin concentration; *POD*, postoperative day; *QoL*, quality of life

## Conclusion

Perioperative iron deficiency and anemia are common in patients presenting for major surgery. Anemia is an independent risk factor for a range of poor postoperative outcomes, including major complications, readmission, and mortality. The definition of anemia should be broadened in the context of major surgery to include women with a hemoglobin concentration of 120–129 g/L. But where the evidence seems settled regarding the *need* to treat preoperative anemia, it remains decidedly unsettled with regarding *how* to treat preoperative anemia, particularly in the setting of functional iron deficiency. Certainly, preoperative anemia management requires careful planning, with the PREVENTT and IVICA trials showing that simply giving a single or divided dose of intravenous iron relatively close to major abdominal surgery does not yield a transfusion benefit, nor improve patient-centered outcomes postoperatively. While clinicians should continue to strive to identify and treat preoperative anemia, further research is required into the timing of this intervention, the groups which benefit most, and the associations between postoperative anemia and poor outcomes.
